# Studies on Electron Escape Condition in Semiconductor Nanomaterials via Photodeposition Reaction

**DOI:** 10.3390/ma15062116

**Published:** 2022-03-13

**Authors:** Chen Ye, Yu Huan

**Affiliations:** 1 School of Chemistry and Chemical Engineering, University of Jinan, No. 336, West Road of Nan Xinzhuang, Jinan 250022, China; yechen-chem@hotmail.com; 2 School of Material Science and Engineering, University of Jinan, No. 336, West Road of Nan Xinzhuang, Jinan 250022, China

**Keywords:** photodeposition, electron escape, semiconductor photocatalyst

## Abstract

In semiconductor material-driven photocatalysis systems, the generation and migration of charge carriers are core research contents. Among these, the separation of electron-hole pairs and the transfer of electrons to a material’s surface played a crucial role. In this work, photodeposition, a photocatalysis reaction, was used as a “tool” to point out the electron escaping sites on a material’s surface. This “tool” could be used to visually indicate the active particles in photocatalyst materials. Photoproduced electrons need to be transferred to the surface, and they will only participate in reactions at the surface. By reacting with escaped electrons, metal ions could be reduced to nanoparticles immediately and deposited at electron come-out sites. Based on this, the electron escaping conditions of photocatalyst materials have been investigated and surveyed through the photodeposition of platinum. Our results indicate that, first, in monodispersed nanocrystal materials, platinum nanoparticles deposited randomly on a particle’s surface. This can be attributed to the abundant surface defects, which provide driving forces for electron escaping. Second, platinum nanoparticles were found to be deposited, preferentially, on one side in heterostructured nanocrystals. This is considered to be a combination result of work function difference and existence of heterojunction structure.

## 1. Introduction

Since the breakthrough development of photocatalysis was reported in 1972 [1], semiconductor material has been studied widely and deeply. These studies not only focused on the invention of new composition, new construction, and functionalized materials but also on the in-depth analysis of their photocatalytic reaction mechanism [2,3,4,5]. The overall semiconductor-driven photocatalytic process includes three steps: (1) the generation of electron-hole charge carriers under the irradiation of a light source; (2) the separation of electrons and holes; (3) the migration of electrons to the reactive sites on the crystal’s surface [6]. Many explanations have been proposed to elucidate the mechanism of the separation of electrons and holes [7]. However, it is difficult to afford direct evidence of the electron motion, despite that an efficient electron escape module could promote the charge carrier separation. Moreover, the photo-induced catalytic reactions will only occur when the electrons come out of the surface. Thus, the studies on how and where the electrons escape from the materials make more sense.

In monodispersed semiconductor nanocrystals, surface engineering is one of the most efficient strategies to overcome the limitations of semiconductor materials. It is practical to improve the properties, as well as application performance of semiconductor nanomaterials, via surface modification and functionalization. Surface engineering of semiconductor materials includes the studies on geometry effects, surface defects, capping ligands, decomposition of nanoparticles or the nanoshell, and so on [8,9,10,11,12,13,14,15,16]. During the last decades, studies on geometry effects on nanocrystal materials have attracted strong interest as a result of the possibility to tailor the materials. Studies on geometry effects of nanomaterials revealed the effects of size, morphology, and surface structure for nanoparticles. Surface geometry effects directly affect the surface properties, including work function and surface energy, which will further affect the particle properties and surface functionalization [13,14,15,16]. Meanwhile, surface defect engineering has also been considered as a useful approach for the modification of electronic and chemical properties of semiconductor nanomaterials, which enhances their activity photocatalysts [17,18,19,20]. Until now, photocatalyst materials, with various types of and abundant defects, have been studied, including metal oxides, metal chalcogenides, graphene materials, etc. [21,22,23,24,25,26]. Some results demonstrated that surface defects may serve as electron traps, making the electrons migrate to a more reactive site, or directly out of the surface [27,28,29,30]. Subsequently, reactions will occur between the surface adsorbed reactants and the escaped electrons. 

Different from monodisperse nanoparticles, heterostructured nanomaterials, which can be formed by loading metal particles or metallic compounds on a surface, are completely other mechanisms. The heterojunction can be a p-n junction or a Schottky barrier. Free electrons existing in these materials were driven to migrate across the junction [31,32,33]. Therefore, the electrons should prefer to escape from the surface of electron attractors such as p-type materials or metal particles. 

In an electrolytic reaction system, only the solid and gas phase products can be easily detected since the solid products will be deposited on the electrode, while gas products can be collected in special vessels. Similar to this, if we intend to observe the electron escape sites on the material surface, the formation of solid products could be a great option. Photodeposition, a method based on the photocatalytic property of semiconductors, is usually used to prepared metal-loading semiconductor materials [34,35,36]. The metal ions, which adsorbed on the surface of semiconductor materials, will react with electrons once they come out from surface. Theoretically, only the metal ions, which adsorbed at or near the electron escaping sites, can be reduced. Furthermore, metal particles will only be deposited at electron escaping sites if the metal ions are small enough. 

In this work, we have studied the electron escaping position via photodeposition of platinum nanoparticles. The electron escaping conditions were identified in different kinds of photocatalyst materials, including the different types of monodisperse semiconductor nanocrystals and the crystals with a special heterojunction structure. The results revealed that surface defects did have good electron trap ability. In addition, the existence of heterojunction also plays an important role for electron motion. 

## 2. Materials & Methods

Chemicals: titanium oxide mix phase nanofiber (TiO_2_ MP, 99%), titanium oxide nanofiber type 1 (TiO_2_ nanofiber type 1, 99%), titanium oxide nanofiber type 2 (TiO_2_ nanofiber type 2, 99%), copper sulfide nanosheets (CuS), star-like BiVO_4_, flower-like Bi_2_WO_6_, and platinum (IV) chloride (PtCl_4_, 98%) were purchased from commercial sources and used without further purification. 

Photodeposition of Pt: the photodeposition of Pt nanoparticles was taken under ultraviolet (UV) light illumination. The photocatalyst materials were dispersed in deionized water and mixed with PtCl_4_ aqueous solution. The mixture, with continuous magnetic stirring, was irradiated with ultraviolet light (λ = 365 nm) from a UV lamp. The light intensity on the sample was 134 mW/cm^2^. For comparison, the reactions without irradiation were also prepared. 

Characterization: transmission electron microscopes (TEM), high-resolution transmission electron microscopy (HRTEM), and selected area electron diffraction (SAED) were carried out on a JEOL 2100F electron microscope (JEOL, Tokyo, Japan), operated with an accelerating voltage of 200 kV. Energy-dispersive X-ray (EDX) analysis and corresponding elemental mapping data were taken with the X-ray spectroscopy (Oxford X-Max 80, Oxford Instruments, Abingdon, UK) attached to the TEM instrument. To prepare the TEM specimens, a drop of nanocrystals, dispersed in ethanol, was dropped on the surface of a lacey formvar/carbon 200-mesh Cu grid. X-ray diffraction analysis (XRD) patterns were collected on a Rigaku SmartlabSE X-ray diffractometer (Rigaku, Tokyo, Japan) by using Cu Kα radiation. 

## 3. Results

**Figure 1:**
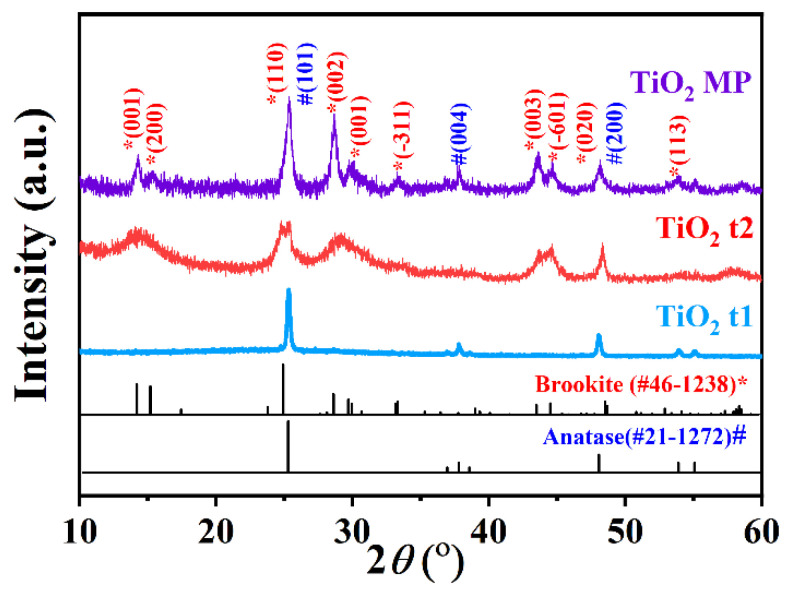
XRD patterns of TiO_2_ MP, TiO_2_ t1, and TiO_2_ t2 samples. The peaks marked by pound sign and asterisk are dependent to anatase phase (JCPDS No. 21-1272) and brookite phase (JCPDS No. 46-1238), respectively.

In this work, the photodeposition of Pt nanoparticles were employed on different kinds of photocatalyst materials, including TiO_2_, Bi_2_WO_6_, BiVO_4_, and CuS. The reaction times and the amount of PtCl_4_ were summarized in Table 1. All the photocatalyst materials in this work were prepared by hydrothermal method. The TiO_2_ nanomaterials used in this work included three types: pure phase TiO_2_ nanofiber type 1 (TiO_2_ t1), pure phase TiO_2_ nanofiber type 2 (TiO_2_ t2), and mixed phase TiO_2_ (TiO_2_ MP). The TiO_2_ MP were synthesized in a one-pot reaction. The crystal structure of the purchased TiO_2_ were characterized by the XRD patterns in Figure 1. It can be observed that the XRD peaks of TiO_2_ t1 coincided well with the brookite phase (JCPDS No. 46-1238), and the XRD peaks of TiO_2_ t2 were indexed to the anatase phase (JCPDS No. 21-1272) without any secondary phase. The broadened XRD peaks should be correlated with the nano-scale particles. The XRD peaks of the TiO_2_ MP sample at 14.2°, 24.9°, 28.6°, 43.5°, and 44.5° were ascribed to the brookite phase, while the peaks at 25.3°, 37.8°, and 48.0° were attributed to the anatase phase. It confirmed that the brookite and anatase phases coexisted in the TiO_2_ MP sample.

**Figure 2:**
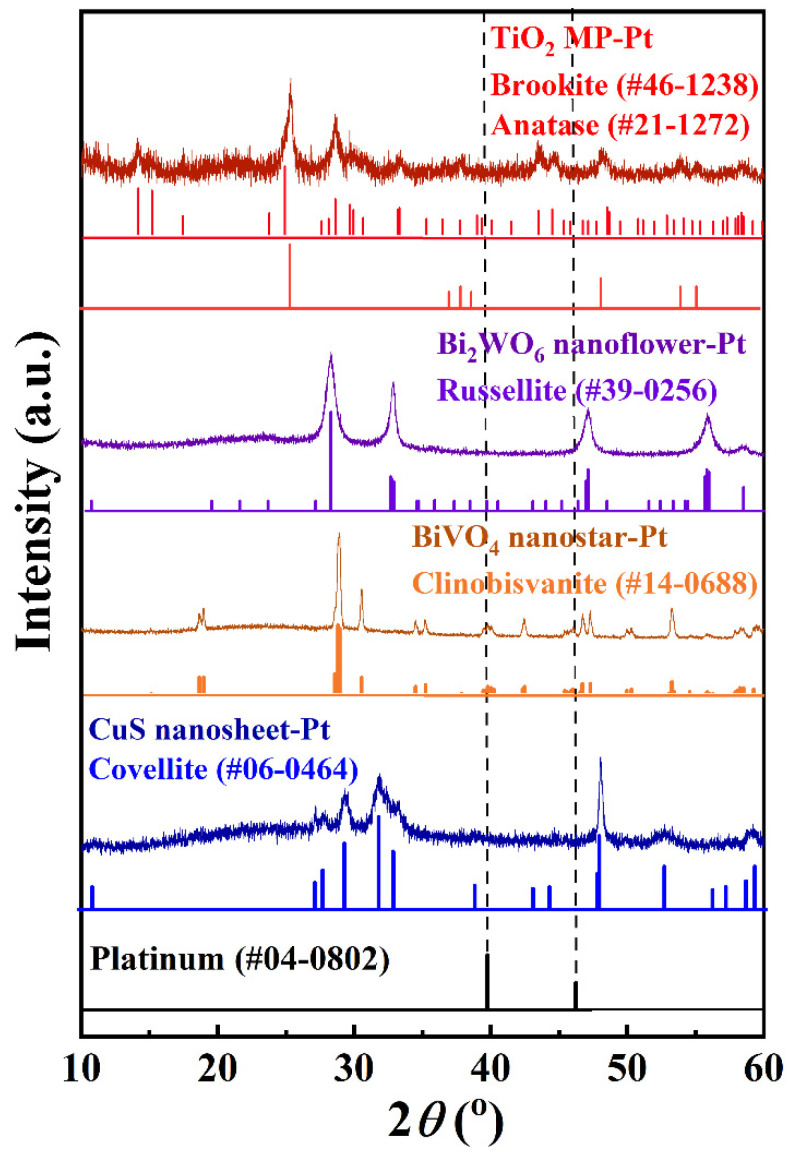
XRD patterns of Pt-deposited TiO_2_ MP nanofiber, Pt-deposited Bi_2_WO_6_ nanoflower, Pt-deposited BiVO_4_ nanostar, and Pt-deposited CuS nanosheet.

Figure 2 showed the XRD results of Pt-deposited Bi_2_WO_6_, BiVO_4_, and CuS photocatalyst materials. The crystal structure of Bi_2_WO_6_, BiVO_4_, and CuS nanomaterials could be illustrated by XRD patterns in Figure 2. The XRD patterns of Bi_2_WO_6_, BiVO_4_, and CuS were well indexed to the russellite phase (JCPDS No. 39-0256), clinobisvanite phase (JCPDS No. 14-0688), and covellite phase (JCPDS No. 06-0464), respectively. These XRD results proved that these nanomaterials had a good crystallization crystal structure and the expected stoichiometric composition. The broadened XRD peaks should be correlated with the nano-scale particles. Since the experiments were attended to load Pt nanoparticles on these photocatalyst materials, typical XRD peaks, located at 39.8° and 46.2° of platinum (JCPDS No. 04-0802), were also marked in the Figure 2. Disappointingly, none of the Pt XRD peaks were detected in these four Pt-deposited photocatalyst materials. This might be because of the small size and low content of Pt nanoparticles. 

**Figure 3:**
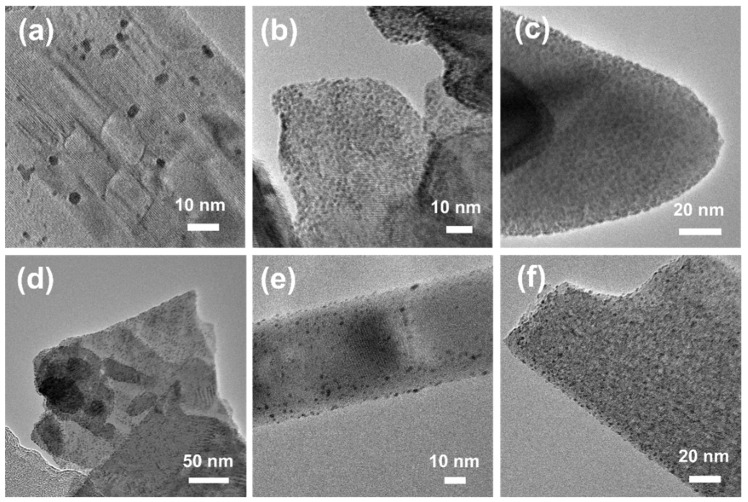
TEM images of Pt-deposited TiO_2_ MP (**a**), Bi_2_WO_6_ (**b**), BiVO_4_ (**c**), CuS (**d**), TiO_2_ t1 (**e**), and TiO_2_ t2 (**f**).

In order to confirm the existing of Pt nanoparticles, TEM, HRTEM, SAED, and EDX analyses were carried out. Figure 3 showed the TEM images of Pt-deposited TiO_2_ MP (a), Bi_2_WO_6_ (b), BiVO_4_ (c), CuS (d), TiO_2_ t1 (e), and TiO_2_ t2 (f). The black dots on the particles, with the size of 1–2 nm, were considered as the Pt nanoparticles. The Pt nanoparticles were homogeneously distributed on the surface of the photocatalyst matrix. In addition, the deposited Pt nanoparticles increased with the increasing PtCl_4_ solution. For instance, the amount of Pt^4+^ used in reactions was 1.2 wt% in TiO_2_-based materials and 2.9 wt% for bismuth and copper compounds in Figure 3. It can also be seen that the amount of Pt particles on bismuth and copper particles were much higher than those on TiO_2_ particles. This result could correspond well with the added amount of PtCl_4_ solution in the reaction system. Therefore, it can be inferred that the 1.2 wt% Pt^4+^ loading amount is not enough for TiO_2_ nanomaterials. In other words, more Pt nanoparticles would be deposited on the surface of TiO_2_ nanomaterials if more PtCl_4_ solution was added during the reaction. 

**Figure 4:**
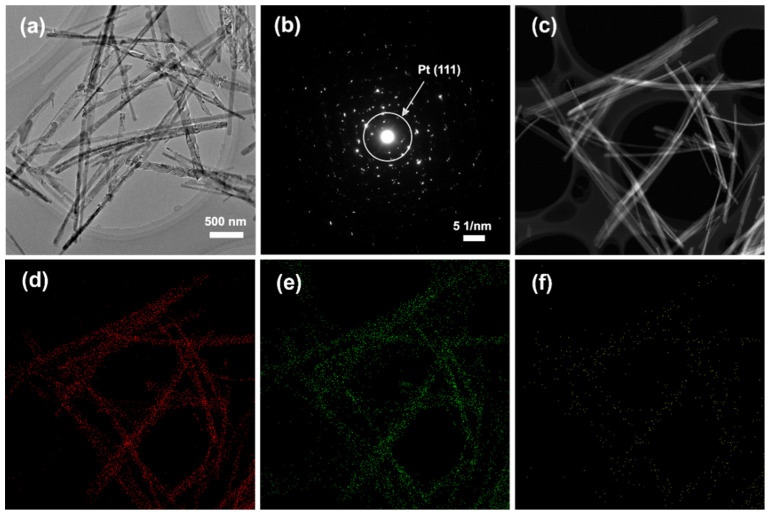
TEM image of Pt deposited TiO_2_ MP (**a**) and the corresponding SAED patterns (**b**); STEM image of Pt deposited TiO_2_ MP (**c**), and the corresponding EDX mapping images of elemental Ti (**d**), O (**e**), and Pt (**f**).

To further verify the existence of the loaded Pt particles, SAED and EDX analyses were applied on the Pt-deposited TiO_2_ nanofibers (Figure 4). In Figure 4b, a pair of diffraction spots, labelled by a red circle with a *d*-spacing of 2.26 Å, was observed. This diffraction data was correlated well with (111) crystal plane of Pt, which finally confirmed the presence of Pt in this Pt-deposited TiO_2_ nanomaterial. Additionally, the scanning TEM (STEM), and the corresponding EDX mapping signal, authenticate uniform distribution of elemental Ti and O throughout the TiO_2_ nanofibers, as well as random distribution of the elemental Pt on the fiber’s surface. 

Above results revealed the successful deposition of Pt nanoparticles on various photocatalyst nanomaterials, including TiO_2_-based, bismuth-based, and copper-based materials; the Pt particles almost occupied the entire surface of core particles. The TEM results also indicated that the Pt particles did not show any selectivity on deposition position; they deposited very randomly. This is considered to be the result of a surface defects-driven electron escape mechanism. In our experiments, only photocatalyst nanoparticles were presented together with the Pt source (PtCl_4_) in aqueous. Under the illumination, electrons generated and migrated to the particle surface to react with adsorbed Pt^4+^ ions. Pt^4+^ ions should be reduced at the electron escaping sites, resulting in the formation of Pt nanoparticle-deposited photocatalyst nanomaterials. As mentioned before, surface defects played important roles for electron migration and escape in monodispersed semiconductor nanoparticles. In general, defects distributed on the surface randomly. This information explained that Pt nanoparticles randomly deposited on semiconductor nanoparticles, which confirmed that the Pt deposition is a result of surface defects engineering. Meanwhile, with different amounts of PtCl_4_ used in the reaction, the amount of Pt nanoparticles deposited on nanofibers changed. As shown in Table 2, the Pt^4+^ loading amount increased from 1.2 wt% to 12.0 wt%, and the Pt-deposited amount increased, linearly, from 0.48 wt% to 7.97 wt%. This could also be evidence that the Pt deposition on monodisperse nanoparticles mainly occurred due the surface defects-driven electron escape mechanism. 

In order to understand the reaction mechanism well, comparison experiments and replenish analyses have been done. Firstly, besides the studies on the precipitate phase, Pt deposited TiO_2_ nanomaterials during the solution phase, which was supposed to contain excess PtCl_4_ and some small photocatalyst nanoparticles, has also been investigated by TEM and EDX analyses. TEM results revealed that there are no free Pt nanoparticles observed, while the EDX mapping signal of Pt has only been detected on residual TiO_2_ nanofibers. Secondly, a comparison experiment, based on a TiO_2_ MP nanofiber sample, was taken in the dark. EDX data shows 0.98 wt% Pt signal. However, the sample prepared with the same condition as the comparison experiment showed a 7.26 wt% Pt signal after illumination for 30 min. Combined with TEM and SAED results, there are neither small particles vision nor a Pt diffraction signal (a d-spacing of 2.26 Å). Such a small amount of Pt loading is regarded as Pt^4+^ ions adsorbed on particle surface. These comparison experiment results further prove the reaction mechanism: Pt^4+^ ions adsorbed on a particle’s surface and reacted with escaped electrons to form Pt nanoparticles, which means Pt nanoparticles were formed and deposited, directly on the particle surface, at electron escaping sites. This mechanism makes it possible to identify electron escaping conditions, directly, by the visual detection of Pt nanoparticles.

**Figure 5:**
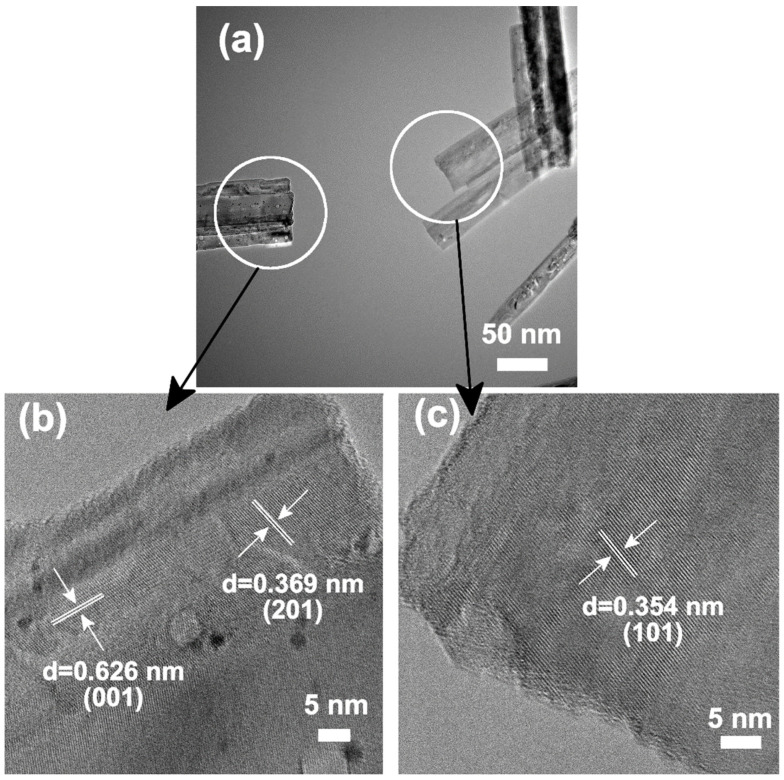
(**a**) TEM images of Pt-deposited TiO_2_ MP sample (with 1.2 wt% Pt^4+^ loading amount). HRTEM images of (**b**) brookite TiO_2_ nanofibers and (**c**) anatase TiO_2_ nanofibers.

Another part of our work was the study on heterostructured TiO_2_ MP nanofibers. As verified by XRD analysis, the TiO_2_ MP sample contained both brookite and anatase phases. The HRTEM images, as shown in Figure 5, also revealed the co-existence of the two phases’ particles in this sample. In the HRTEM picture of the left nanofiber (Figure 5b), two lattice fringes, with *d* spacing 6.26 Å and 3.69 Å, were correlated well with that of the (001) and (201) planes of the brookite phase (JCPDS No. 46-1238), respectively. For the right counterpart (Figure 5c), lattice fringes, with a *d* spacing 3.54 Å, could be indexed to the (101) planes of the anatase phase (JCPDS #21-1272). The HRTEM results were in accordance with the XRD analysis. 

Interestingly, the Pt nanoparticles preferred to be reduced on the brookite TiO_2_ nanofibers instead of the anatase TiO_2_ nanofibers, which could be seen from Figure 5a. As analyzed in the above TEM results (Figure 3e,f), in pure TiO_2_ t1 and t2 samples, most of the particles show obvious Pt deposition in both brookite and anatase TiO_2_ nanofibers when 1.2 wt% PtCl_4_ was added. Considering that the Pt nanoparticles were only photodeposited at the electron escape site, the electron escape sites should exist in both brookite and anatase TiO_2_ nanofibers. However, when the same amount of PtCl_4_ was used to react with TiO_2_ MP nanoparticles, a different phenomenon was observed. In the mixed phase sample, Pt nanoparticles were deposited on partial nanofibers. By analyzing TEM, it seems that Pt nanoparticles were preferred to deposit on brookite phase nanofibers (Figure 5b) compared to anatase fibers (Figure 5c), when the Pt^4+^ loading amount is 1.2 wt%. This can also be evidenced by the EDX analysis data in Table 2. Compared to the pure t1 and t2 samples, mixed phase TiO_2_ nanofibers show almost the same Pt deposition amount. This means the Pt nanoparticles were mainly deposited on one kind of TiO_2_ nanomaterial instead of not being deposited. Furthermore, this phenomenon became more obvious when the Pt^4+^ loading amount decreased to 0.70 wt% and even to 0.23 wt%. By studying a relatively low Pt^4+^ loading amount sample, we were able to distinguish these particles more evidently, with or without loading Pt particles. It confirmed that nanofibers with the brookite phase showed higher reaction activities for the photocatalytic metal deposition compared to anatase phase particles. This can be attributed to the difference of work function. Referring to Vera and co-workers’ results [37], work function of brookite TiO_2_ is a little bit smaller than in the anatase phase. Although work function value is variable for different testing methods, in different testing environments, or even based on different facets, work function of the brookite phase is always smaller than anatase under the same conditions. EDX analysis results also present the same trend, in which pure TiO_2_ t2 samples have a higher Pt deposited amount. 

**Figure 6:**
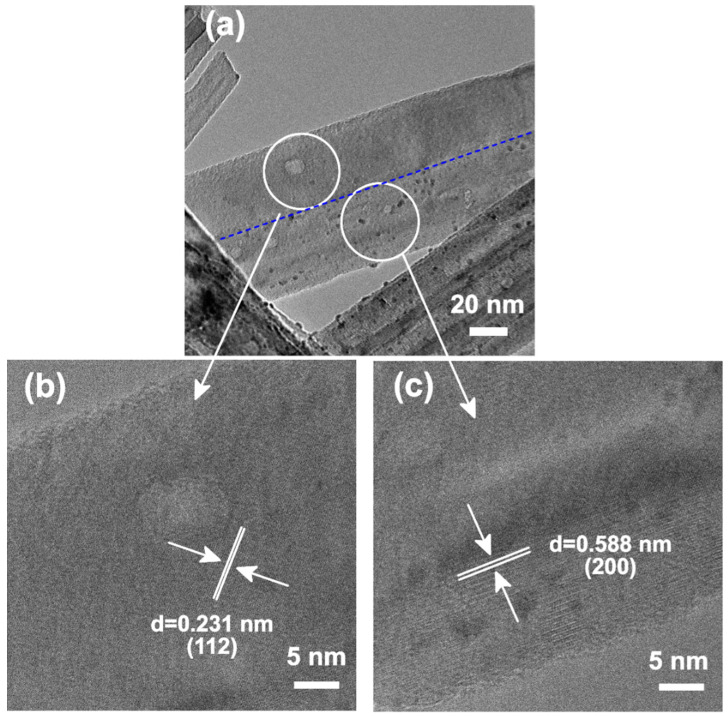
TEM images of a nanoparticle with a heterostructure (**a**) in the Pt-deposited TiO_2_ MP sample (with 1.2 wt% Pt^4+^ loading amount). HRTEM images of (**b**) upper and (**c**) lower areas, as indicated in (**a**).

Except for the two kinds of pure phase particles, a two phase co-existence structure was fabricated side by side on one single particle. In Figure 6, a heterostructure TiO_2_ nanofiber, with brookite and anatase phases, was observed. It is obvious that a distinct interface between the two phases was observed, in one particle, by the bright-field TEM image. Figure 6b showed the high resolution image of the upper side, in which the lattice fringes, with a *d* spacing 2.31 Å, was identified. This area was correlated with (112) the crystal plane of the anatase TiO_2_ phase. The bottom part, with *d* spacing 5.88 Å lattice fringes, has been decided as the brookite phase. TEM results showed that the two phases co-existed, and a special heterostructure containing the brookite and anatase phases was constructed in one particle. 

Meanwhile, TEM results also revealed that almost all the Pt nanoparticles are loaded on the bottom part, which is the brookite phase. This phenomenon declared that the electrons mainly escaped from the surface of the brookite TiO_2_. As discussed previously, there are two effects that could be used to explain this phenomenon. First, some heterostructure materials have an electrical junction that could promote the separation of the charge carrier and drive the electrons across the junction. In this mixed phase sample, the electrons mainly escaped from the brookite phase. Thus, it is inferred that the brookite phase was performed as an electron attractor component. The existence of heterojunction efficiently causes the migration of electrons across the junction interface, and then, they come out from the surface of the counterpart with the electron attractor component. It resulted in the higher content of escaped electrons from brookite TiO_2_ particles compared with the pure brookite TiO_2_ sample. Therefore, the Pt nanoparticles, attached on the brookite TiO_2_ particles in the heterojunction sample, were more than that in the pure brookite TiO_2_ sample. Second, the different work functions of these two phases would be other driving forces for the different Pt deposition densities. 

**Figure 7:**
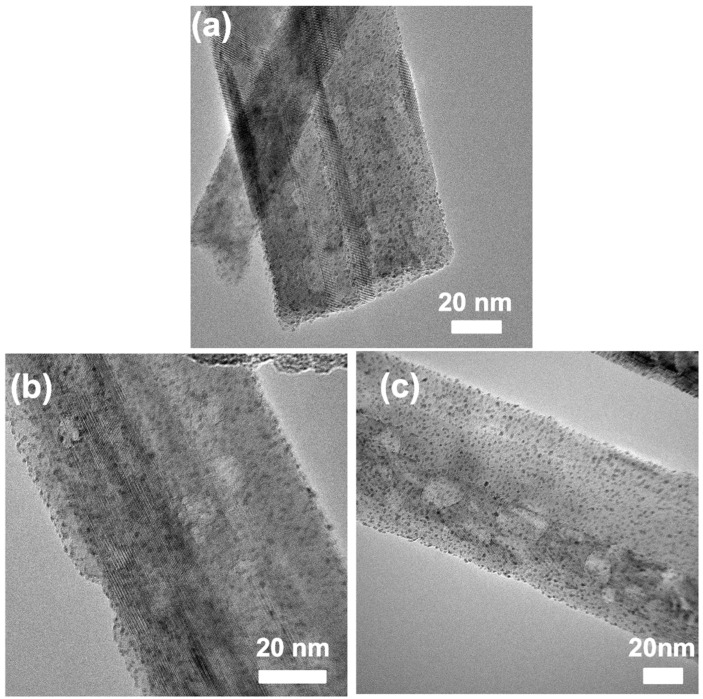
TEM images of Pt-deposited TiO_2_ MP nanofibers with Pt^4+^ loading amount 2.3 wt% (**a**), 5.8 wt% (**b**), and 12.0 wt% (**c**), respectively.

Figure 7 shows the TEM images of Pt-deposited TiO_2_ MP nanofibers with a Pt^4+^ loading amount from 2.3 wt% to 12.0 wt%. From Figure 7a,b, a clear viewer of the different Pt deposited densities, on two sides of the heterostructures, was observed even if the Pt^4+^ loading amount increased to 5.8 wt%. The work functions, different between brookite phase (around 3.72 eV) and anatase phase (around 3.84 eV) [37], may not be big enough to result in such different Pt-deposited densities, especial at higher Pt^4+^ loading amounts. Thus, we believe that both the heterojunction driven force and work function difference contribute to the Pt deposited density difference between brookite and anatase phases in heterostructured nanoparticles. In Figure 7c, Pt nanoparticles abundantly deposited on both sides, which could be attributed to the saturation of active sites on the brookite component surface. 

From another point of view, the Pt deposit sites directly point out the electron escape site. In monodisperse nanomaterials, Pt nanoparticles are loaded randomly. This attributes to the abundant surface defects of photocatalyst materials. Surface defects are considered as the main electron escape driving force. Different from this, in some heterostructure materials, electrons are driven to migrate to the electron attractor component, due to the existence of heterojunction structure and the difference of work functions. Thus, although Pt^4+^ ions are already adsorbed on the entire surface, only those ions located at the electron escape side are successfully reduced. Our results correspond well with those.

## 4. Conclusions

In summary, electron escaping conditions of various kinds of photocatalyst materials have been studied through photodeposition reactions. For photocatalyst nanomaterials, which show monodisperse crystal structure, Pt nanoparticles deposited randomly on the surface. This is attributed to the rich surface defects. In the heterostructured photocatalyst nanomaterials, Pt nanoparticles are preferred to load at one side. This is due to the electron migration driving force provided by heterojunction structure and work function difference. This is considered as a higher force, to promote the separation and migration of electrons, than the one caused by surface defects. Our results correspond well with known conclusions published previously. This is enough to demonstrate that photodeposition is usable for the study of electron escaping conditions. Further, it can be used as a “tool” to identify the catalytic ability of photocatalyst materials—especially the activity of their single particle.

## Figures and Tables

**Table 1:** Summary of the crystal structure of Pt deposited nanocrystals and the reaction conditions.

Basic Materials	Crystal Structure	Pt^4+^ Loading Amount	Reaction Time
TiO_2_ t1	Brookite	1.2–12.0 wt%	30 min
TiO_2_ t2	Anatase	1.2–12.0 wt%	30 min
TiO_2_ MP	Brookite + Anatase	0.23–12.0 wt%	15 min–2 h
Bi_2_WO_6_	Russellite	2.9 wt%	30 min
Bi_2_VO_4_	Clinobisvanite	2.9 wt%	30 min
CuS	Covellite	2.9 wt%	30 min

**Table 2:** Summary of Pt^4+^ loading amounts and actual Pt-deposited amounts, analyzed by EDX, for TiO_2_-based materials.

Basic Materials	Pt^4+^ Loading Amount	Pt Deposited Amount (Analyzed by EDX)
TiO_2_ t1	1.2 wt%	0.76 wt%
	2.3 wt%	2.05 wt%
	5.8 wt%	4.07 wt%
	12.0 wt%	7.96 wt%
TiO_2_ t2	1.2 wt%	0.31 wt%
	2.3 wt%	3.15 wt%
	5.8 wt%	6.03 wt%
	12.0 wt%	8.70 wt%
TiO_2_ MP	1.2 wt%	0.37 wt%
	2.3 wt%	2.30 wt%
	5.8 wt%	4.20 wt%
	12.0 wt%	7.26 wt%

## Data Availability

The data presented in this study are available on request from the corresponding author.
